# Circulating Biomarker Panorama in HIV-Associated Lymphoma: A Bridge from Early Risk Warning to Prognostic Stratification

**DOI:** 10.3390/biom15070993

**Published:** 2025-07-11

**Authors:** Xuejiao Shu, Qing Xiao, Yi Liu, Ya Li, Xiaoqing Xie, Sanxiu He, Jun Li, Xiaomei Zhang, Yao Liu

**Affiliations:** 1 School of Medicine, Chongqing University, Chongqing 400030, China; 202437021038@stu.cqu.edu.cn (X.S.); 202237021003@stu.cqu.edu.cn (Y.L.); 2016110342@stu.cqmu.edu.cn (Y.L.); 2 Chongqing Key Laboratory for the Mechanism and Intervention of Cancer Metastasis, Department of Hematology-Oncology, Chongqing University Cancer Hospital, Chongqing 400030, China; 2016110112@stu.cqmu.edu.cn (Q.X.); 2018100811@cqu-edu.cn (X.X.); 2018110456@hospital.cqmu.edu.cn (S.H.); skiplijun888@alu.scu.edu.cn (J.L.)

**Keywords:** HIV-associated lymphoma (HAL), non-Hodgkin’s lymphoma (NHL), HIV, biomarker, cytokines, chemokines, microbial translocation, extracellular vesicles (EVs), miRNA

## Abstract

HIV-associated lymphoma (HAL) is a heterogeneous and highly aggressive group of malignancies. Although antiretroviral therapy (ART) has significantly prolonged the survival of people living with HIV (PLWH), the risk of malignancy secondary to HIV infection remains higher than in HIV-negative individuals, with HAL being among the most frequent. The pathogenesis of HAL is complex, involving multifactorial interactions. In current clinical practice, HAL faces a double challenge: the lack of effective biological risk warning systems and the lack of precise prognostic stratification tools. In recent years, the construction of multidimensional biomarker systems has shown critical value in the comprehensive management of HAL. This review aims to systematically summarize recent advances in circulating biomarkers for HAL, focusing on the potential applications of immune environment indicators, such as inflammatory cytokine profiles and microbial translocation markers, as well as serum protein profiles, lymphocyte subsets, extracellular vesicles (EVs), circulating microRNAs (miRNAs), and viral biomarkers. These biomarkers offer promising avenues for early risk prediction, therapeutic monitoring, and prognostic evaluation. Developing an assessment system based on multidimensional biomarkers will optimize early risk stratification, enable precise prognostic classification, and support personalized therapeutic strategies, thereby providing a novel theoretical basis and practical direction for the clinical management of HAL.

## 1. Introduction

The disease burden of human immunodeficiency virus (HIV) infection and its oncologic complications remains a major global public health challenge. According to the latest epidemiologic data from the Joint United Nations Programme on HIV/AIDS (UNAIDS), as of 2023, an estimated 39.9 million adults worldwide were living with HIV, and approximately 630,000 deaths were attributed to AIDS-related illnesses [1]. Although the widespread application of antiretroviral therapy (ART) has substantially extended the survival of people living with HIV (PLWH), the incidence of malignancies, particularly AIDS-defining cancers (ADCs) [2,3], continues to rise and has become one of the leading causes of death among PLWH [4,5,6]. Given the highly aggressive nature of HAL, recent research has increasingly focused on building early risk warning systems. Studies have shown that before HAL is clinically diagnosed, PLWH often exhibit characteristic biological alterations in the blood, such as elevated levels of B-cell stimulatory factors (e.g., cytokines and chemokines). This immune activation profile is closely associated with an increased risk of lymphoma development, suggesting that these biomarkers could serve as potential indicators for the early detection of HAL [7,8,9]. These findings also offer a potential window for targeted intervention during the subclinical phase of the disease.

Moreover, due to the considerable variability in HAL prognosis, accurate prognostic evaluation is equally critical [10]. Although prognostic models such as the International Prognostic Index (IPI) and the age-adjusted International Prognostic Index (aaIPI) are widely used in clinical practice [11], they insufficiently account for HIV-specific factors and do not incorporate HIV-related biomarkers, limiting their ability to precisely stratify high-risk patients with poor outcomes [12,13,14]. Thus, there is an urgent need to develop next-generation prognostic models that integrate clinical features with molecular and immunological biomarkers to guide individualized treatment decisions. Against this background, identifying novel biomarkers for early risk prediction, therapeutic monitoring, and prognostic evaluation has become a major focus for optimizing HAL management. Circulating abnormalities in inflammatory mediators, immune activation factors, and microbial translocation-related molecules are now recognized as key features associated with HAL development and poor outcomes.

This review systematically summarizes recent advances in circulating biomarkers in HAL, covering inflammatory molecules, microbial translocation markers, lymphocyte subsets, extracellular vesicles, miRNAs, immunoglobulin (Ig), and viral-related markers, and explores their translational potential for risk stratification, therapeutic monitoring, and prognostic assessment, providing theoretical support for the development of precision intervention strategies aimed at improving outcomes in PLWH.

## 2. Epidemiological Overview

Non-Hodgkin’s lymphoma (NHL) is one of the most common types of ADCs [15]. It includes diffuse large B-cell lymphoma (DLBCL), Burkitt lymphoma (BL), primary plasmoblastic lymphoma (PBL), and primary effusion lymphoma (PEL), according to the latest World Health Organization (WHO) classification [16]. Epidemiological data indicate that over 90% of HAL cases are classified as NHL [17]. Historical data from the United States show that, before the introduction of ART, the risk of developing NHL among PLWH was 113 times higher compared to HIV-negative individuals [18]. This phenomenon likely contributed to the significant increase in overall NHL cases observed in the United States during the 1980s [19]. Recent meta-analyses have demonstrated that even in the ART era, PLWH continue to have an approximately 23-fold higher risk of developing NHL compared to the general population [20]. As the most common fatal cancer among PLWH, HIV-associated NHL (HIV-NHL) accounts for more than one-third of all HIV-related cancer deaths [4,21,22], a trend confirmed by multiple large cohort studies [23,24,25]. The prognosis for HIV-NHL remains poor. Studies have reported that approximately 34% of patients with HIV-NHL die within the first year after diagnosis [26], with a two-year overall survival rate of around 58% [27] and a five-year survival rate of only 55% [26]. These data underscore the ongoing challenges in the clinical management of HAL and highlight the urgent need for improved early detection, risk stratification, and treatment strategies. Comparatively, Hodgkin lymphoma (HL), as a non-AIDS-defining cancers (NADCs), has a substantially lower overall incidence than NHL. However, studies have shown that PLWH remain at a significantly higher risk of developing HL compared to the general immunocompetent population, with marked variations in incidence observed across different countries and between sexes [28]. In recent years, advancements in treatment have led to a continuous improvement in 5-year survival rates among HL patients [29,30], though outcomes remain suboptimal in resource-limited settings [31]. Given that this review focuses primarily on NHL, the discussion of HL is limited to a brief overview.

## 3. Pathogenesis

**Figure 1:**
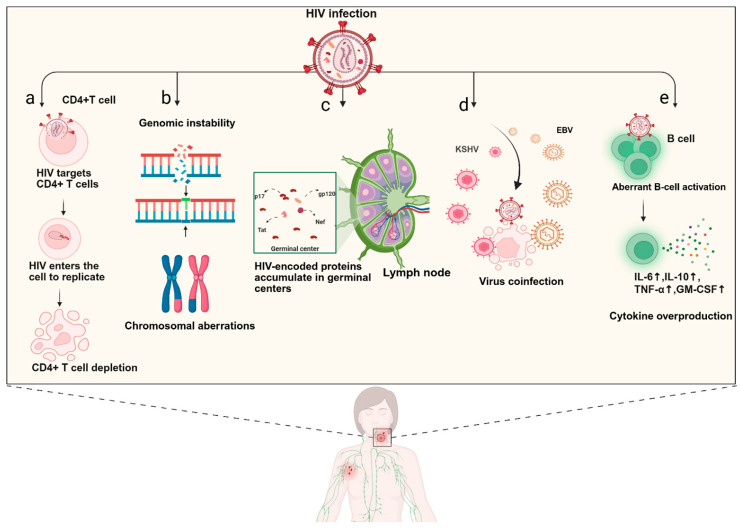
The pathogenesis of HIV-associated lymphoma (HAL). (**a**) HIV infection targets the CD4+ T cells, entering to replicate internally, leading to cell depletion and eventually triggering a severe immunodeficiency in the body that drives lymphomas; (**b**) HIV infection destabilizes the genomic instability, leading to chromosomal aberrations, DNA recombination, and ultimately driving lymphomagenesis; (**c**) HIV itself and various HIV-encoded proteins, including p17, nef, tat, gp120, act directly on follicular germinal center aggregates in lymph nodes to drive lymphomas; (**d**) impaired immune surveillance of T cells after HIV infection increases the chances of tumorigenesis driven by oncogenic viruses such as EBV and KSHV, leading to lymphomagenesis; (**e**) HIV infection causes chronic antigen activation and B-cell activation, long-term chronic inflammation, and the overproduction of various cytokines that promote the development and growth of lymphoma. The solid arrows (↑) represent elevated. Created in BioRender. Xuejiao Shu. (2025) https://app.biorender.com/illustrations/67f232faa3efd15ebf9d87de.

The high morbidity and mortality of HAL are closely linked to its unique pathological mechanisms, which involves multifactorial interactions that have not yet been fully elucidated [32,33]. Current mainstream views suggest that the following mechanisms are involved: (a) HIV infection leads to CD4+ T-cell depletion, resulting in severe immunodeficiency [34]; (b) genomic instability and genetic aberrations, which predispose people to malignant transformation [35]; (c) the direct oncogenic effects of HIV-encoded viral proteins, such as p17, gp120, transactivator of transcription (Tat), and negative factor (Nef) [36]; (d) the synergistic oncogenic effects of opportunistic infections, notably Epstein–Barr virus (EBV) and Kaposi’s sarcoma-associated herpesvirus (KSHV, also known as human herpesvirus 8) [37,38]. In addition, chronic antigenic stimulation and aberrant B-cell activation associated with HIV infection, along with persistent overproduction of cytokines, also play significant roles in the pathogenesis of HAL [39] (Figure 1). Our recent studies have revealed that the highly aggressive nature of HAL may be attributed to malignant B cells’ reliance on oxidative phosphorylation for energy metabolism and to pronounced tumor immune escape mechanisms. Key features include the loss of major histocompatibility complex class I (MHC-I) expression, T-cell exhaustion-like functional impairment, and macrophage-mediated immunosuppressive microenvironment [40].

## 4. Plasma Metabolite Changes

**Figure 2:**
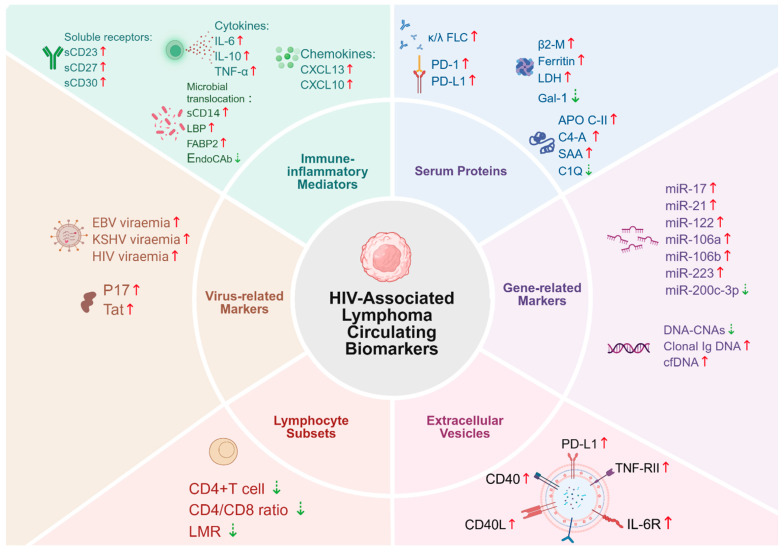
Panoramic overview map of circulating biomarkers for HAL. This figure categorizes six types of circulating biomarkers involved in the onset, progression, and prognosis of HAL. Red solid arrows (↑) indicate upregulation, while green dashed arrows (⇣) indicate downregulation. Created in BioRender. Xuejiao Shu. (2025) https://app.biorender.com/illustrations/68103ee84139ebc329581dce.

Due to its accessibility and minimally invasive nature, blood is widely used for biomarker screening and validation in precision oncology medicine [41]. The development of HAL is closely associated with alterations in various plasma metabolites. In recent years, studies have demonstrated that serum cytokines, chemokines, soluble receptors, and serum proteins play crucial roles in the early stages of disease progression, risk prediction, and prognostic evaluation. Moreover, emerging molecular biomarkers such as circulating miRNAs, extracellular vesicles (EVs), and clonally rearranged Ig DNA have further expanded the research landscape of plasma metabolite changes. Immunological dysfunction, persistent HIV viremia, and sustained expression of HIV-encoded viral proteins together contribute to altered plasma metabolic profiles, thereby promoting tumor microenvironment (TME) remodeling and immune evasion. Figure 2 categorizes and presents six classes of circulating biomarkers associated with the occurrence, progression and prognosis of HAL.

## 5. Immunoinflammation-Related Biomarkers

### 5.1. Cytokines

Cytokines play important roles in regulating immune responses, development, metabolism and tumorigenesis. Interleukin (IL)-6, IL-10, and tumor necrosis factor-alpha (TNF-α), as immunomodulatory cytokines widely distributed in the human body, are involved in the acute-phase response of the organism and anti-infective immunity by affecting antibody production and cell proliferation and differentiation [42,43,44,45]. Abnormal expression of these cytokines has been closely associated with malignant lymphoid neoplasms, promoting B-cell proliferation, genomic instability, and lymphoma initiation and progression, while also serving as indicators of poor prognosis [46,47,48,49].

In PLWH, genetic variation in IL-10 and its specific genotypes has potential value in HIV-NHL risk identification. Studies have shown significantly increased IL-10 expression and a higher frequency of the 592C/C genotype in the promoter region among HIV-NHL patients [50]. Further genetic analyses have revealed subtype-specific associations of IL-10 single nucleotide polymorphisms (SNPs), where the rs1800896_G allele correlates with increased systemic HIV-NHL risk, while the rs1800871_T and rs1800872_A alleles are associated with a reduced risk of primary central nervous system lymphoma (PCNSL) [51]. These findings suggest that genetic variations in IL-10 regulatory regions influence expression levels, and high-expressing IL-10 genotypes could serve as genetic risk markers for individualized risk stratification based on anatomical lymphoma subtypes. In addition, multicenter cohort studies have also shown a time-dependent increase in the levels of cytokines such as IL-6 and IL-10 before NHL diagnosis in PLWH, independently of CD4^+^ T-cell counts [52]. Combined testing for other cytokines such as IL-1α, IL-4, IL-5, IL-13, and granulocyte-macrophage colony-stimulating factor (GM-CSF) can also help identify people at high risk for HAL [9]. In animal experiments, HIV-1 transgenic Tg26 mice, a model for studying HIV-associated diseases [53], exhibit progressive elevation of multiple cytokines (e.g., IL-1β, IL-6, IL-10, TNF-α, IL-12p40, and IL-13, and GM-CSF in plasma, closely associated with B-cell lymphoma development [54]. This is consistent with the trend in HAL patients. Some studies have further confirmed that sustained elevations of cytokines including IL-6, IL-10, interferon gamma-induced protein 10 (IP-10)/CXCL10, TNF-α, and neopterin 1–5 years before HIV-NHL diagnosis are positively correlated with disease risk [55,56]. Moreover, combined biomarker models incorporating IL-11, IL-18, IL-29, and CXCL11 have shown superior predictive performance compared to single markers [7,57], highlighting the necessity of multiplex cytokine profiling. These cytokines are also markedly elevated in HIV-associated HL (HIV-HL) [58], suggesting their pivotal roles in HAL pathogenesis and their potential utility for early prediction and diagnosis (Table 1).

In terms of prognosis, high levels of IL-6, IL-10, and B-cell-activating factor of the TNF family/B lymphocyte Stimulator (BAFF/BLyS) have been associated with poorer overall survival (OS) and progression-free survival (PFS) in HIV-NHL patients [59,60]. However, the independent prognostic value of individual cytokines remains somewhat inconsistent across studies. After R+EPOCH therapy (rituximab plus etoposide, vincristine, doxorubicin, cyclophosphamide, and prednisone), although IL-6 and IL-10 levels showed no significant overall change, elevated IL-6 was significantly associated with shortened OS, and high IL-10 with reduced PFS [59]. In HIV-associated DLBCL (HIV-DLBCL) patients, elevated IL-10 and transforming growth factor-β (TGF-β) levels were independently associated with poor OS and PFS, regardless of IPI scores, while IL-6, despite being highly expressed, did not correlate with prognosis [61]. These discrepancies may stem from cohort heterogeneity, lymphoma subtype differences, and variations in treatment regimens (e.g., R+EPOCH versus R±CHOP). Nevertheless, cumulative evidence supports a strong link between cytokine dysregulation and unfavorable HAL outcomes. In addition to the factors mentioned above, IL-7, IL-8, IL-15, and interferon-γ (IFN-γ) were also strongly associated with increased risk of disease-free survival (DFS) and death; particularly, IL-7 and IL-8 were also strong independent predictors of DFS and OS respectively [62].

In summary, cytokines are not only important risk markers for the development of HAL but also hold potential as indicators for prognostic assessment. Jointly monitoring changes in specific cytokine levels prior to disease onset could facilitate early screening of high-risk individuals and the implementation of targeted interventions to improve clinical outcomes. However, given the variability in the predictive value of cytokines across different lymphoma subtypes, future studies should not only expand sample sizes but also consider constructing stratified predictive models based on histological subtypes (e.g., HIV-DLBCL and PCNSL) to enhance the specificity and clinical applicability of cytokine-based biomarkers. In addition, further research is warranted to explore the potential of targeting cytokine-related pathways (such as IL-6R inhibitors) in improving patient prognosis.

### 5.2. Chemokines

Chemokines are a family of secreted proteins with molecular weights ranging from 8–12 kDa, classified into CXC, CC, CX3C, and C chemokine subfamilies based on the arrangement of their N-terminal cysteine residues [63]. In the TME, chemokines participate in pathogenesis through dual mechanisms. On the one hand, they guide specific immune cells (such as B cells, T cells, and macrophages) to inflammatory sites via chemotactic gradients to regulate local immune responses. On the other hand, they act directly on tumor cells and endothelial cells to activate proliferative signaling pathways, maintain cancer stem cell phenotypes, and promote epithelial-to-mesenchymal transition (EMT), thereby driving malignant progression [64]. Consequently, the chemokine network serves as a crucial bridge linking tumor immunity to clinical outcomes [64,65,66].

CXCL13 (also known as B-cell-attracting chemokine 1, BCA-1) plays a vital role by interacting with its receptor CXCR5 to direct B-cell trafficking to secondary lymphoid organs such as the spleen and lymph nodes [67,68,69]. Aberrant activation of the CXCL13/CXCR5 axis has been implicated in the pathogenesis of various non-HIV-associated B-cell lymphoma subtypes [70,71,72,73,74,75]. In PLWH, dysregulation of CXCL13 may disrupt B-cell migration and damage lymphoid architecture, thereby promoting HIV-NHL development [76,77]. Several clinical studies have demonstrated the potential value of CXCL13 in the early diagnosis of HIV-NHL [56,78] and have shown significantly elevated serum CXCL13 levels as early as 2.5 years before lymphoma diagnosis [79]. The correlation between CXCL13 and systemic HIV-NHL risk was even more significant [7]. Additionlly, genetic factors may also influence CXCL13 expression and HIV-NHL risk. Specifically, the rs355689 variant in the CXCL13 gene is associated with reduced risk through decreased CXCL13 expression, whereas the rs630923 variant in CXCR5 correlates with increased CXCL13 levels and heightened lymphoma risk [56]. Regarding prognosis, HIV-NHL patients treated with R-EPOCH who had lower baseline CXCL13 levels were more likely to achieve complete remission, while those with higher levels exhibited significantly shortened OS and PFS [59], supporting the role of CXCL13 as a prognostic biomarker and a potential tool for individualized therapy guidance.

Another important chemokine is CXCL10, also known as IP-10, a 10-kDa secreted polypeptide belonging to the CXC family [63]. Previous studies reported that elevated CXCL10 levels are associated with rapid HIV disease progression [80,81,82,83]. In the field of HIV-NHL, early studies found significantly higher circulating CXCL10 levels in HAL patients compared to PLWH without lymphoma [84]. However, this study was limited by the small sample size and the concentration of specimen collection within one year prior to the diagnosis of lymphoma, making it difficult to clarify whether elevated CXCL10 is an early marker of lymphomagenesis or a secondary phenomenon close to the diagnostic period. Vendrame and colleagues found that sustained elevation of CXCL10 in the three-time windows prior to HIV-NHL diagnosis (>3 years, 1–3 years, and 0–1 year) increased the risk of morbidity by more than 3.7-10-fold, and high CXCL10 levels correlated with adverse prognosis significantly [55]. These findings were further validated in independent cohorts and AIDS Malignancy Consortium trials, supporting CXCL10 as both an early biomarker and a predictor of poor outcomes in HIV-NHL [57,60].

Other chemokines such as CCL2 and CCL17 also play significant roles in HIV-NHL. In immunocompetent DLBCL patients, high serum levels of CCL2 are associated with poorer OS and PFS [85]. Under HIV-infected conditions, elevated serum CCL2 and CCL17 levels correlate strongly with increased HIV-NHL risk [7]. Data from animal models also support the predictive value of CCL2 and macrophage inflammatory protein-1β (MIP-1β) for disease progression [54]. Importantly, CCL2 levels partially normalize following therapeutic interventions, suggesting a potential role as a dynamic prognostic marker [60]. CCL8, CCL19, CXCL11, and MIP-1δ have also shown abnormal expression in HIV-NHL [57]. Their potential involvement in immune cell trafficking and immune regulation points to a possible role in HIV-NHL pathogenesis. However, current evidence remains insufficient, and their clinical relevance requires validation in larger cohorts.

In conclusion, chemokines, by modulating the immune microenvironment and tumor behavior, serve multifaceted roles in the early warning, mechanistic understanding, and prognostic evaluation of HIV-NHL (Table 2). Some chemokines have shown promising potential for clinical translation. Nevertheless, for chemokines with less established roles, further large-scale studies are necessary to confirm their clinical utility. Future research should integrate multi-omics approaches and dynamic monitoring strategies to elucidate the temporal evolution of the chemokine network and its clinical implications.

### 5.3. Soluble Receptors

CD23, a low-affinity Fc receptor for IgE, is primarily expressed on the surface of B cells, and its expression is positively correlated with B-cell activation, serving as an important marker of B-cell activation [86]. The membrane-bound form of CD23 can be proteolytically cleaved to generate soluble CD23 (sCD23), which further promotes B-cell proliferation through a positive feedback mechanism [87,88]. Elevated serum sCD23 levels have been associated with the staging and prognosis of certain B-cell malignancies, such as chronic lymphocytic leukemia [89,90], suggesting that sCD23 may play a role in the pathological regulation of malignant B cells. Within the tumor necrosis factor receptor (TNF-R) superfamily, CD27 and CD30 are key co-stimulatory molecules mainly expressed on memory B cells [91,92]. Their soluble forms, sCD27 and sCD30, are generated by proteolytic cleavage and serve as biomarkers of immune activation [93,94]. Previous studies have demonstrated that sCD27 levels are elevated in HIV infection [95] and other conditions of immune dysregulation [96,97], which is informative for its use in HIV-NHL studies.

The Martínez-Maza’s group has systematically revealed the predictive value of serum sCD23, sCD27, and sCD30 in HIV-NHL [7,52,78,98,99,100]. These markers showed stronger associations with systemic HIV-NHL risk, particularly among individuals who had not received ART [52,78]. It has been observed that sCD23 is more strongly correlated with PCNSL than with systemic HIV-NHL [7], a finding consistent with earlier reports by Bossolasco [101]. This difference may reflect variations in pathological subtypes or blood-brain barrier permeability across study populations.

sCD163, a cleavage product of the macrophage scavenger receptor CD163, has also emerged as a marker of interest [102]. Elevated sCD163 levels are positively associated with HIV-NHL risk in a dose-dependent manner, especially in PCNSL [7], suggesting that it may be involved in virus-driven tumorigenesis, particularly EBV-associated PCNSL [103]. Previous studies in HL have similarly linked sCD163 with EBV-DNA presence [104]. In terms of therapeutic monitoring, recent clinical data showed that sCD25 levels significantly decreased after R-EPOCH treatment, while sCD44 and sCD163 levels remained unchanged [60]. sCD163 may not be useful for monitoring treatment response, dynamic changes in sCD25 could serve as a potential indicator of therapeutic efficacy.

In summary, combined detection of sCD23, sCD27, and sCD30 offers promising potential for ultra-early risk stratification in HIV-NHL, while sCD163 may serve as a specific diagnostic marker for EBV-driven subtypes, particularly PCNSL. Additionally, dynamic monitoring of sCD25 could complement therapeutic response evaluation. These soluble receptors not only deepen our understanding of HIV-NHL pathogenesis but also provide a foundation for biomarker-driven, stratified management strategies (Table 3). Future multicenter prospective studies are warranted to validate their predictive utility and clarify their mechanistic roles in lymphomagenesis.

### 5.4. Microbial Translocation Biomarkers

The gut microbiota maintains host immune homeostasis through dynamic interactions among epithelial cells, immune cells, and commensal bacteria [105]. Under physiological conditions, host barrier systems effectively prevent microbial products from entering the circulation [106]. However, HIV infection compromises gut barrier integrity, increasing permeability and allowing microbial products such as lipopolysaccharides (LPS) to translocate into the systemic circulation [106,107,108,109]. Even under ART, gut barrier restoration remains incomplete, and persistent microbial translocation becomes a major driver of systemic immune activation and inflammation in PLWH [108,109,110].

**Figure 3:**
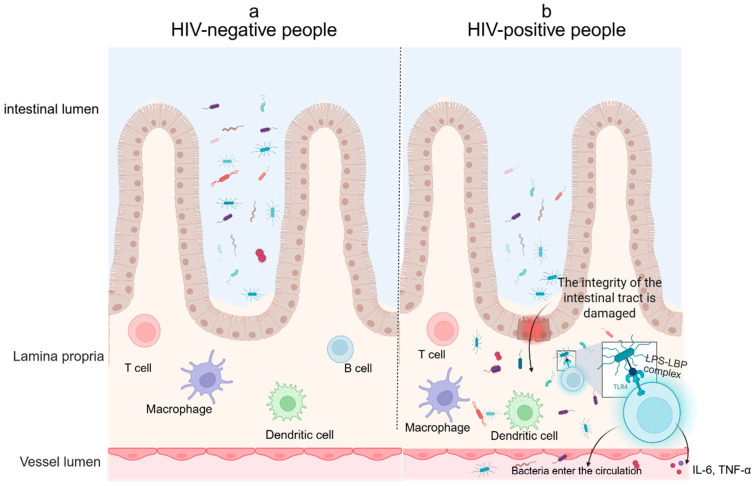
The disruption of intestinal barrier integrity and microbial translocation in HIV-positive individuals. (**a**) In HIV-negative individuals, an intact gut epithelial barrier effectively prevents microbial products from entering the circulation; (**b**) HIV infection damages the gastrointestinal epithelium, leading to increased permeability and allowing bacterial products such as LPS to translocate into the lamina propria and bloodstream. LPS forms complexes with LBP, which activate the TLR4/NF-κB signaling pathway and trigger the release of inflammatory cytokines including IL-6 and TNF-α, promoting lymphomagenesis.Created in BioRender. Xuejiao Shu. (2025) https://app.biorender.com/illustrations/67f68ca3b9e5bb8ed54bd2ff.

sCD14, a specific marker of monocyte/macrophage activation, is released via phospholipase-mediated membrane shedding and vesicle secretion [111]. LPS, a key component of Gram-negative bacterial outer membranes, can translocate into the systemic circulation following gut barrier disruption [112]. LPS binds to lipopolysaccharide-binding protein (LBP) and sCD14 to form a complex that activates the Toll-like receptor 4 (TLR4) signaling pathway, leading to NF-κB–mediated secretion of proinflammatory cytokines such as IL-6 and TNF-α [107,108,111,113]. Serum fatty acid-binding protein 2 (FABP2) levels serve as a marker of gut epithelial damage [114,115]. Endotoxin core antibody (EndoCAb), which neutralizes circulating LPS, reflects mucosal barrier integrity [116]; its reduction indicates microbial overstimulation and is associated with immune overactivation and poor prognosis [107,117]. The bioactivity of LPS is dynamically regulated by a network involving LBP, sCD14, and EndoCAb [118]. Thus, in PLWH, gut barrier dysfunction leads to increased microbial translocation, stimulating monocyte/macrophage activation and sustained systemic inflammation, which promotes lymphomagenesis [110,117] (Figure 3).

Elevated levels of sCD14, LBP, FABP2, combined with decreased EndoCAb, form a “microbial translocation signature” that has been validated as a biomarker for disease monitoring [119]. Given the close association between chronic immune activation and HIV-NHL, microbial translocation markers have attracted considerable attention. Persistent elevation of sCD14, LBP, LPS, FABP2, and haptoglobin, along with decreased EndoCAb, has been linked to increased HIV-NHL risk [7,8]. Notably, sCD14 shows a stronger association with central nervous system (CNS) HIV-NHL, while FABP2 and LBP are more related to systemic HIV-NHL [7]. Recent studies have extended the prognostic relevance of these markers. Baseline sCD14 levels are significantly inversely correlated with OS in HIV-NHL patients, and dynamic reductions in sCD14 and LBP following R-EPOCH therapy may indicate a partial reversal of immune activation, providing additional prognostic information [60].

In summary, microbial translocation biomarkers offer significant potential for risk prediction, prognostic stratification, and treatment response evaluation in HIV-NHL (Table 4). Although the precise molecular mechanisms linking microbial translocation to lymphomagenesis require further elucidation, these markers provide a strong theoretical basis for biomarker-guided stratified management strategies. Future research should aim to clarify the interactions between microbial dysbiosis, host immunity, and tumorigenesis, and explore targeted interventions focused on restoring gut barrier integrity. Additionally, given that HIV infection alters the composition of the intestinal flora, changes in fecal flora in patients with HAL warrant further exploration to help develop targeted intestinal strategies.

## 6. Protein-Related Biomarkers

### 6.1. Immunoglobulins

Immunoglobulin consists of two homologous heavy (H) and light (L) chains linked by disulfide bonds. Based on the heavy chain type, they are classified into five major classes: IgG, IgA, IgM, IgD, and IgE. Light chains exist as either kappa (κ) or lambda (λ) isotypes, with each chain containing a variable domain and multiple constant domains [120]. During B-cell development, H and L chains combine to form a complete immunoglobulin, while excess light chains are secreted into the blood as free light chains (FLCs) [121]. The clonal characteristics of FLCs and the κ/λ ratio have become important biomarkers reflecting abnormal B-cell activation, with significant applications in the diagnosis, therapeutic monitoring, and prognosis evaluation of cancers and inflammatory diseases [122,123,124,125]. In recent years, FLCs as a marker of B-cell activation have gained attention for their predictive value in HAL. However, the available findings remain heterogeneous regarding the optimal predictive time window. Several studies have demonstrated that serum κ and λ FLC levels significantly increase within five years before the diagnosis of HIV-NHL [55,126]. Although prediagnostic elevation is consistently observed, the precise temporal window for optimal predictive accuracy remains uncertain. Notably, FLCs appear to retain predictive value throughout this this five-year period in individuals with CD4 counts ≥100 cells/mm^3^, whereas among those with CD4 counts <100 cells/mm^3^, the association is primarily evident 2–5 years before diagnosis [126]. Shepherd et al. also reported that FLCs showed better predictive performance when measured more than two years before HIV-NHL diagnosis [127,128]. In contrast, findings from an Italian cohort demonstrated that κ+λ FLC levels exceeding 1.5 times the normal upper limit within two years before HAL diagnosis were associated with a sharply increased risk, with the strongest predictive performance observed during this period [129]. Although FLCs show certain advantages as early risk warning biomarkers, discrepancies in predictive timing have been reported, which may be influenced by multiple factors, including CD4+ T-cell counts, viral load, and lymphoma subtypes. Moreover, the prognostic relevance of FLCs in HAL remains unclear, as current studies have not identified a significant association [61].

Compared with FLCs, the role of total serum immunoglobulin levels in HIV-NHL risk prediction remains controversial. Early studies suggested that elevated serum globulin levels were associated with increased HIV-NHL risk [130], while later larger cohort studies failed to confirm this association [131], possibly due to differences in study design, sample characteristics, detection methods, and the impact of widespread ART use. Additionally, conventional immunoglobulin subtypes such as IgA, IgE, IgG, and IgM have been reported to be not significantly associated with the risk of HIV-NHL [52,126,127].

### 6.2. PD-1 and PD-L1

Programmed cell death protein 1 (PD-1), an important immunosuppressive molecule, is a member of the immunoglobulin superfamily [132]. PD-1 and its ligand programmed cell death ligand 1 (PD-L1) are key immune checkpoint molecules that regulate immune responses. They maintain immune tolerance by suppressing T-cell function and limiting the overreaction of the immune system [133]. However, this pathway is also exploited by various malignant tumor cells to evade immune surveillance and promote tumor progression [134]. In recent years, immune checkpoint inhibitors targeting the PD-1/PD-L1 pathway have shown significant efficacy in the treatment of several malignancies [133]. In HAL, the overexpression of PD-1/PD-L1 is not only closely related to immune escape mechanisms but may also play a crucial role in tumor initiation and progression. Epeldegui et al. found that the proportion of PD-L1+ B cells was significantly elevated in PLWH, and this phenomenon could be detected several years before the lymphoma diagnosis [135]. These PD-L1+ B cells predominantly exhibit a regulatory B-cell (Breg) phenotype, capable of secreting IL-10 and interacting with T cells through the PD-1/PD-L1 axis to suppress their immune function, thereby providing a favorable microenvironment for lymphoma immune escape. Moreover, in EBV-positive HIV-NHL patients, high expression of PD-1 has also been confirmed to be closely associated with poorer OS and PFS, suggesting that PD-1 is not only a marker of immune escape but also an independent prognostic factor [136].

In the context of HIV infection, chronic immune activation may further induce sustained high expression of PD-1/PD-L1, thus promoting tumor development and progression. Therefore, PD-1/PD-L1 not only holds potential as a biomarker for predicting HAL risk but also provides a theoretical basis for the clinical application of immune checkpoint inhibitors in such patients. Joint monitoring of PD-1 and PD-L1 expression could improve early screening efficiency for high-risk individuals and provide a foundation for personalized immunotherapy strategies.

### 6.3. Gal-1

Galectin-1 (Gal-1), a β-galactoside-binding protein, plays a critical role in immune regulation [137]. In HL, increased Gal-1 expression has been associated with poor prognosis and is considered a biomarker for disease progression [138]. In HAL, Gal-1 exhibits distinct expression patterns. Serum Gal-1 levels are lower in PLWH, particularly among those who subsequently develop HAL, whereas intratumoral Gal-1 expression is relatively elevated and has been associated with a better prognosis [139]. This discrepancy may be explained by changes in immune status and disease progression. HIV infection alters the host immune system, potentially suppressing the secretion of Gal-1 by immune cells and resulting in lower serum Gal-1 levels. In contrast, intratumoral Gal-1 expression appears to be independently regulated, with tumor cells and cells within the TME capable of autonomously producing Gal-1. High intratumoral Gal-1 expression may represent an adaptive response during tumor development, promoting tumor growth, immune evasion, and angiogenesis. Although current research remains limited, Gal-1 shows promising potential in the HAL field and warrants further in-depth investigation.

### 6.4. β2-Microglobulin

β2-microglobulin (β2-M) is a classic marker of tumor burden [140,141], and its serum levels are significantly associated with disease staging in HIV-negative DLBCL. Although some studies have suggested that β2-M alone may have limited diagnostic utility in HIV-NHL [142], recent clinical data indicate that elevated serum β2-M is directly associated with increased tumor cell proliferation and tumor burden [143]. These findings support its role as a biomarker for lymphoma progression and poor prognosis. Additionally, elevated β2-M levels have also been reported at the time of HIV-HL diagnosis [144]. These observations suggest that β2-M holds potential for risk prediction and prognostic assessment, although its utility may vary across different HAL subtypes. Further large-scale studies are needed to validate these findings.

### 6.5. Ferritin

Serum ferritin is recognized as an important prognostic factor in cancer detection [145], with its levels closely associated with tumor burden and disease activity in hematologic malignancies [146]. In immunocompetent patients with DLBCL, elevated ferritin levels are significantly correlated with poor prognosis [147,148], and the combination of ferritin with the IPI further enhances prognostic stratification [149]. Similarly, elevated ferritin levels are significantly associated with reduced survival, independently of IPI scores and CD4^+^ T-cell counts in HIV-DLBCL [61]. Furthermore, studies have suggested that ferritin levels may be linked to the polarization status of tumor-associated macrophages and could reflect the TME, offering potential guidance for rituximab-based therapy [150]. Overall, these findings underscore the significant prognostic value of ferritin as an independent biomarker in HAL.

### 6.6. Lactate Dehydrogenase

Serum lactate dehydrogenase (LDH) is one of the most classic biomarkers reflecting tumor burden and metabolic activity, and its elevation is significantly associated with poor prognosis in hematological malignancies [151,152]. In clinical practice for HAL, LDH has become an important independent prognostic indicator due to its accessibility, cost-effectiveness, and strong association with inferior PFS and OS [153,154,155]. However, its independent predictive value, threshold definitions, and clinical significance vary across different subtypes. In HIV-DLBCL, most studies support a strong correlation between LDH levels and OS, particularly when LDH exceeds twice the upper limit of normal (2 × ULN), where the 2-year OS rate significantly declines [156,157,158]. The prognostic value of LDH for PFS remains controversial. Some studies have reported that elevated LDH shortens median PFS by approximately four months but fails to retain independent predictive power [159], while more recent larger cohort studies confirmed LDH as an independent predictor of PFS [158]. These differences across studies may be related to the immune status of the study population or the evolution of treatment regimens. In contrast, in BL, the prognostic value of LDH is even more pronounced. When LDH exceeds 5 × ULN, both 3-year PFS and OS are significantly reduced [160]. In the latest study by Liu et al., LDH levels above 3 × ULN were significantly associated with PFS but required an even higher threshold to impact OS [161]. Such threshold differences suggest underlying biological heterogeneity among study populations, highlighting the need to optimize risk stratification and therapeutic strategies based on disease subtype. Multicenter studies are needed to establish standardized thresholds.

Additionally, LDH holds prognostic value in other special HAL subtypes. In HIV-associated PBL (HIV-PBL), LDH levels above 300 U/L significantly shortened median OS [162]. In HIV-associated PCNSL (HIV-PCNSL), elevated cerebrospinal fluid (CSF) LDH levels were correlated with tumor progression [163], providing a potential tool for disease monitoring in this unique setting. Recently, Liang’s group [164] integrated LDH and other variables to develop a new prognostic model, which outperformed the traditional IPI system in HIV-DLBCL prognostication, offering a feasible stratification approach for resource-limited settings, pending external and multicenter validation. In conclusion, LDH remains a biomarker of significant clinical value in HAL prognosis. Combined detection with other emerging biomarkers may further enhance the precision of prognostic assessment, necessitating ongoing translational research efforts.

### 6.7. Other Biomarkers

Proteomics provides an efficient tool for exploring HAL biomarkers, and it has demonstrated unique advantages in high-throughput protein screening, disease mechanism analysis and drug target development [165,166]. Detection of serum protein mass spectrometry reveals a number of differentially expressed proteins, such as apolipoprotein C-II (Apo C-II), complement C4-A, and serum amyloid A (SAA), which have important roles in the TME and are closely associated with the risk of HAL development [167]. Notably, elevated SAA levels have also been correlated with prolonged survival in HAL patients [168]. These differentially expressed proteins may assist in the early identification of high-risk PLWH, enabling closer monitoring and early intervention. Regarding prognostic evaluation, levels of the complement C1Q complex are closely associated with disease progression in HIV-NHL patients, and a decrease in C1Q levels after treatment predicts poor prognosis [142].

In summary, the detection of these serum protein biomarkers provides new molecular targets for the early diagnosis and prognostic evaluation of HAL, and holds promise for advancing individualized treatment strategies (Table 5). These molecules, through their dynamic expression patterns, enable the early identification of high-risk PLWH and show significant associations with TME remodeling and treatment response. It is important to note that the applicability of traditional biomarkers, such as β2-microglobulin and LDH, may vary across different HAL subtypes, suggesting that biomarker selection and application should be optimized according to disease-specific characteristics in clinical practice. Future research should build upon multicenter and external validation studies to further integrate proteomic data and develop multi-omics predictive models, ultimately enhancing the accuracy of prognostic assessment and the level of personalized therapy for HAL patients.

## 7. Genetic-Related Biomarkers

### 7.1. MiRNAs

MiRNAs are a class of endogenous, non-coding single-stranded RNAs, typically 18–24 nucleotides in length. Each miRNA can target multiple messenger RNAs (mRNAs), negatively regulating gene expression by promoting mRNA degradation or inhibiting translation [169,170,171]. Due to their tissue-specific expression patterns and high stability, miRNAs can be easily extracted from blood and other body fluids, making them promising candidates as diagnostic biomarkers and prognostic tools for cancer [172,173,174]. In recent years, the critical role of miRNAs in tumor biology has attracted increasing attention. Aberrant miRNA expression profiles have been recognized as common features across various malignancies, where they may function either as oncogenes or tumor suppressors [175,176]. In solid tumors, the regulatory networks involving miRNAs have been gradually elucidated [177,178,179]. Similarly, in hematological malignancies, dysregulation of miRNAs has been shown to be closely associated with the pathogenesis of lymphomas [180,181] and leukemias [182,183].The development of normal B cells is tightly regulated by miRNAs, and disruption of this regulation is considered a major driving factor in lymphomagenesis [184,185].

In HAL, miR-21 as an oncogenic miRNA shows significant elevation several years before diagnosis and can be used as a marker for early risk prediction [186,187,188]. In contrast, the high expression of miR-122 and miR-223 was more significantly associated with the risk of developing HIV-DLBCL and PCNSL [187]. The miR-17-92 cluster and its related miRNAs (such as miR-17, miR-106a, and miR-106b) are also markedly overexpressed in HIV-NHL, likely promoting tumor progression through negative regulation of the cell cycle inhibitor p21 [189].

While most studies have focused on the overexpression of miRNAs, recent investigations have also addressed miRNA downregulation. In animal models, miR-200c-3p was found to be significantly downregulated in HIV-1-treated BL cells [190], leading to the upregulation of its target proteins ZEB1 and ZEB2, which subsequently promote cancer cell migration and invasion [191,192]. These findings suggest that HIV-1 infection may accelerate lymphoma progression and metastasis by reshaping miRNA regulatory networks, highlighting the importance of early miRNA-based screening in high-risk HAL populations. However, whether downregulation patterns provide comparable predictive utility remains to be systematically validated. In addition, genetic studies have identified specific SNPs, such as DDX20 rs197412, miR-196a2 rs11614913, and HIF1A rs2057482, that are associated with an increased risk of HIV-NHL [193], which provides a genetic rationale for individualized risk stratification for screening.

In a word, current evidence strongly supports the role of miRNAs in HAL and potential targets for risk stratification, and the value of circulating miRNAs as an important biomarker for the early detection of HAL should not be ignored. However, issues such as disease specificity of miRNA expression and the impact of ART on miRNA profiles require further exploration. Future studies should aim to develop more precise miRNA detection methods, elucidate the specific regulatory networks involved, and refine individualized risk prediction and therapeutic strategies.

### 7.2. Circulating DNA

In recent years, studies have revealed that HIV-NHL possess distinct molecular and immunogenomic characteristics compared to those in HIV-negative individuals. M.I. Tiirikainen et al. found that in immunocompromised HIV-positive hosts, the progression of DLBCL requires fewer somatic genomic alterations. Tumor samples from HIV-positive patients exhibit fewer regional DNA copy number alterations (CNAs), and the frequency of CNAs is significantly lower than that observed in HIV-negative individuals [194]. In HAL, Trine Engelbrecht Hybel et al. identified multiple gene mutations, most commonly in KMT2D, TNFAIP3, and TP53. However, no subtype-specific gene variants were identified, indicating substantial genetic heterogeneity among different histological subtypes of HAL [195]. Moreover, the immunogenomic landscape of HIV-NHL is profoundly influenced by EBV status. In EBV-negative patients, T cells retain their ability to recognize tumor-specific neoantigens, particularly those related to IgH rearrangements, offering potential avenues for T-cell-based immunotherapy [196].

At the molecular level, under physiological conditions, B lymphocytes generate diverse antibody repertoires through random recombination of Ig gene segments [120]. In contrast, malignant transformation of B cells typically results in clonal Ig gene rearrangement, a hallmark of B-cell lymphomas [197,198]. In recent years, various high-throughput sequencing technologies have been applied to the study of circulating free DNA (cfDNA), particularly tumor-derived circulating tumor DNA (ctDNA). Clonal Ig gene rearrangements can be detected in the cfDNA of patients with NHL or HL [199,200,201,202,203], and such rearrangements are frequently observed in PLWH diagnosed with lymphoma [204]. The presence of Ig DNA rearrangement in plasma serves as a lymphoma-specific tumor biomarker (Table 6); patients who fail to clear clonal Ig DNA after treatment may be at high risk of failure with standard therapy [204]. The correlation between plasma cIg levels and both lymphoma disease burden and treatment response suggests that clonal Ig could serve as a sensitive and specific biomarker [202,205]. A more recent study [206], used next-generation sequencing (NGS) to detect plasma cfDNA in patients with HIV-associated aggressive B-cell lymphoma, finding clonal Ig heavy chain sequences in 55% of cases. In contrast, no such sequences were detected in HIV-infected patients with concomitant tuberculosis, further supporting the disease specificity of this biomarker.

**Table 6:** Genetic-related biomarkers.

Biomarker Category	Representative Biomarkers	Sample Source	Expression Trend	Clinical Relevance	References
miRNAs	miRNA SNPs miR-17 miR-21 miR-122 miR-106a miR-106b miR-223 miR-200c-3p	HIV-NHL patients HIV-NHL patients HIV-NHL patients HIV-NHL patients HIV-NHL patients HIV-NHL patients HIV-NHL patients HIV-BL cell line	Increased Increased Increased Increased Increased Increased Increased Decreased	Predictive Predictive Predictive Predictive Predictive Predictive Predictive Predictive	[193] [189] [186,187,188] [187] [189] [189] [187] [190]
Circulating DNA	DNA-CNAs Clonal Ig DNA cfDNA	HIV-NHL patients HIV-NHL patients HIV-NHL patients	Decreased Increased Increased	Predictive Predictive/Treatment Monitoring Predictive	[194] [204] [206]

Abbreviations: miRNAs, microRNAs; HIV-NHL, HIV-associated non-Hodgkin’s lymphoma; HIV-BL, HIV-associated Burkitt lymphoma; SNPs, single nucleotide polymorphisms; DNA-CNAs, copy number alterations; Ig, Immunoglobulin; circulating free DNA.

## 8. Extracellular Vesicles

EVs are membrane-bound structures secreted by cells, capable of carrying nucleic acids, proteins, and metabolites [207]. EVs can specifically express immune regulatory molecules such as PD-L1 and CD40 ligands on their surface, facilitating intercellular communication and modulation of the TME through ligand–receptor interactions, thereby promoting tumor progression [208,209,210,211]. EVs have attracted significant attention in the field of cancer liquid biopsy [208,212]. In immunocompetent NHL patients, circulating EVs have been validated as biomarkers for diagnosis and prognostic assessment [213,214]. In the context of HAL, EVs carrying immune regulatory molecules such as PD-L1, CD40, CD40L, and TNF receptors-II (TNF-RII) are significantly elevated and correlate with levels of inflammatory cytokines [215]. Baseline levels of EVs expressing PD-L1 or IL-6R can independently predict the risk of HIV-NHL, with TNF-RII+ EVs being particularly associated with the DLBCL subtype [216].

Overall, EVs represent a promising emerging class of biomarkers for early risk prediction and therapeutic monitoring in HIV-NHL (Table 7). The specific molecules they carry may also directly regulate disease progression. Furthermore, EV-associated miRNAs, proteins, and DNA offer considerable potential for liquid biopsy applications. However, studies investigating EVs in HAL remain limited, and several challenges persist, including small sample sizes, unclear mechanistic insights (particularly in the HIV setting), and technical barriers in EV isolation. Future research should focus on expanding cohort sizes, clarifying the molecular mechanisms of EVs in HAL, and optimizing isolation and detection techniques to facilitate their clinical translation.

## 9. Lymphocyte Subpopulation

### 9.1. CD4+ T Cells and CD4/CD8 Ratio

CD4+ T cells are the primary targets of HIV infection. Their decline, along with a reduced CD4/CD8 ratio, reflects immune dysfunction and has been widely used to assess immune status and lymphoma risk in PLWH. Studies have confirmed that lower CD4+ T-cell counts and CD4/CD8 ratios not only sensitively reflect immune reconstitution but also serve as independent predictors for the development of ADCs [217]. Regarding predictive value, earlier studies suggested that the historical nadir of CD4+ T-cell counts was closely associated with HAL risk [218,219]. In mid to late ART, more studies have highlighted that recent CD4+ T-cell counts are stronger independent predictors [131,220,221,222,223]. A study from the UK National HIV Oncology Centre demonstrated that while CD4+ T-cell counts were generally lower at HAL diagnosis in the pre-ART era, significant immune recovery was observed following the widespread introduction of ART, with higher CD4+ T-cell counts at lymphoma diagnosis thereafter [224]. This suggests that once CD4+ T-cell counts are restored under ART, the impact of prior immunodeficiency on NHL risk diminishes [225], and greater emphasis should be placed on current immune status. Recent advancements in dynamic immune monitoring have enabled comparisons across baseline, nadir, and current CD4+ T-cell counts, as well as CD4/CD8 ratios. It has been found that low current CD4+ T-cell counts and a CD4/CD8 ratio <0.4 are independent predictors of HIV-NHL [226,227]. In the United States, a CD4/CD8 ratio <0.3 significantly increased NHL risk and demonstrated long-term predictive value [228], consistent with findings from Spanish cohorts [229]. Moreover, an international study across Europe and Australia indicated that a CD4/CD8 ratio <0.5 independently predicted HIV-NHL [230]. However, Caby et al. based on a French multicenter cohort proposed different conclusions, as they found that the CD4/CD8 ratio did not show a statistical association with the risk of HIV-NHL in PLWH with ≥500/mm^3^ CD4+ T cells [231]. Such discrepancies may be attributable to variations in baseline immune status, timing of measurements, methodologies, or lymphoma subtype distributions.

In terms of prognosis, low CD4+ T cells are associated with inferior OS and PFS among HAL patients [232,233]. A CD4/CD8 ratio <0.5 is also an independent predictor of non-AIDS-related and all-cause mortality among PLWH, including those with NHL [234]. Our center’s data further demonstrate that newly diagnosed HAL patients exhibit significantly reduced CD4+ T cells and CD4/CD8 ratios, accompanied by elevated CD8+ T-cell counts, IL-6, and IL-2R levels—all factors associated with poor prognosis [235]. Additionally, S.K. Barta’s team integrated CD4+ T-cell counts, viral load, and AIDS history into a HAL-specific prognostic model, which outperformed the traditional aaIPI for OS prediction and risk stratification, although its performance for predicting CR and PFS remained limited [236]. Combining the CD4/CD8 ratio with IPI further improves prognostic accuracy and offers practical guidance for therapeutic decision-making in HAL [237].

### 9.2. Lymphocyte-to-Monocyte Ratio

The lymphocyte-to-monocyte ratio (LMR) reflects the extent of systemic inflammatory responses in cancer patients and has demonstrated prognostic value in both solid tumors and HIV-negative diffuse large B-cell lymphoma [238,239]. Zeng et al. were the first to validate its applicability in HIV-DLBCL [240]. Higher LMR (≥2) was significantly associated with longer survival in HAL [241]. Our center has recently developed an ARDPI model that includes LMR and CD4/CD8 ratio. Compared with IPI, the ARDPI model has higher predictive accuracy and accurately predicts survival in newly diagnosed HIV-DLBCL patients [14]. However, the prognostic value of LMR in other HIV-associated aggressive lymphoma subtypes remains to be further validated.

Overall, CD4+ T-cell counts, CD4/CD8 ratio, and LMR have become important biomarkers for risk assessment and prognostic prediction in HAL (Table 8). A persistently low CD4/CD8 ratio suggests a high risk of HAL development, and its combination with traditional indices such as the IPI can further enhance the accuracy of risk prediction and prognostic stratification. Future studies should focus on large-scale, multicenter prospective cohorts to determine the optimal timing and cutoff values for these biomarkers. In clinical practice, it is recommended to strengthen lymphoma screening for PLWH with a persistently inverted CD4/CD8 ratio and to incorporate emerging prognostic models into therapeutic decision-making and follow-up monitoring.

## 10. Virus-Related Biomarkers

### 10.1. HIV Viraemia

HIV viremia contributes to the increased risk of HAL not only by inducing CD4+ T-cell depletion and chronic immune activation but also through the direct oncogenic mechanisms of HIV-1 itself [242,243,244]. Studies have shown that cumulative HIV viraemia is an independent predictor of HAL development [223]. PLWH with HIV RNA levels exceeding 10^5^ copies/mL exhibit a significantly higher risk of developing HIV-NHL [131,221,245]. However, the quantification of cumulative viremia effects varies across regions. In the German study, it was found that for every 2000 days log_10_ copies/mL increase in cumulative viremia was associated with a 67% higher overall lymphoma risk, with the greatest risk observed in the BL subtype [246]. In contrast, studies from the United States focused on the impact of low-level viremia, showing that HIV RNA levels between 51–500 copies/mL increased the risk of HIV-NHL by 66–110%, with DLBCL being the most strongly associated subtype [247]. Multivariate analyses further indicated that for each 1 log_10_ copies/mL increase in HIV RNA, the risk of NHL increased by 24–42% [247,248]. These findings suggest a clear dose-dependent relationship between HIV viremia levels and HIV-NHL risk.

By comparison, some studies have shown that although high HIV viral loads prior to ART initiation increase the risk of lymphoma and other cancers, early initiation of ART can substantially mitigate this risk. Consequently, the association between HIV viremia and lymphoma risk becomes less pronounced during effective ART treatment [249,250]. Notably, persistent viremia even after HIV-NHL diagnosis has been associated with increased mortality [251], underscoring the importance of maintaining strict viral suppression during chemotherapy.

Animal model studies have revealed that although ART effectively reduces plasma viral load, it is powerless to remove viral reservoirs in the lymph nodes, and the virus can rebound rapidly after stopping the drug [252]. Future HIV cure strategies must therefore focus on targeting these hidden reservoirs within deep tissues such as lymph nodes. Additionally, in humanized mouse models, the use of caspase-1 inhibitor VX-765 significantly reduced immune activation, CD4+ T-cell depletion, plasma viremia, and the establishment of HIV-1 reservoirs [253]. These findings suggest that early ART initiation combined with anti-inflammatory therapy could effectively limit reservoir formation. Furthermore, in patients initiating ART during the AIDS phase, the proviral integration sites showed monoclonal expansion, which progressively intensified over time. This clonal expansion reduces immune system heterogeneity, potentially increasing susceptibility to infections and malignancies, including lymphoma. These observations offer mechanistic insights into the elevated risk of opportunistic infections and cancers among long-term survivors [254].

### 10.2. HIV-Encoded Proteins

The prevailing view is that the development of HAL is closely related to the sustained action of HIV-encoded proteins. These viral proteins can drive oncogenic processes through multiple mechanisms, with their sustained presence forming a molecular foundation for cumulative risk [255]. Among them, the Tat and p17 matrix proteins are considered pivotal contributors to HAL development. Tat promotes lymphomagenesis by inducing immune evasion [256], oxidative stress and DNA damage [257], chromosomal translocations and genomic instability [258,259,260,261], dysregulation of signaling pathways and transcriptional networks [260,262], as well as compensatory responses and adaptive evolution [263]. These processes establish a vicious cycle of “immune escape–genomic instability–aberrant proliferation” that ultimately drives lymphoma development. Similarly, p17 promotes malignant transformation by activating key signaling cascades such as PI3K/Akt and MEK/ERK, inducing abnormal B-cell proliferation, genomic instability, and remodeling of the TME [54,264,265,266]. Studies have shown that p17 is highly detectable in plasma and tissues [267,268], even in patients with a good response to ART therapy. Compared to other HIV proteins (such as gp120, p24, nef, and tat), p17 is already highly expressed during the early stages of lymphoma, whereas the latter proteins tend to rise during the later stages [54,264,269]. Moreover, natural variation in the *p17 gene* during HIV-1 infection has been shown to differentially regulate intracellular signaling pathways and promote clonal expansion of B cells, thereby accelerating lymphomagenesis [270]. Specific *p17 genevariants* further enhance B-cell clonal growth and increase the risk of HAL development [271,272].

To conclude, persistent HIV viremia and the continuous expression of HIV-encoded proteins jointly contribute to the development and progression of HAL. Cumulative viral load shows a dose-dependent association with lymphoma risk, emphasizing the need for dynamic monitoring and sustained viral suppression to minimize oncogenic stimulation and improve outcomes. In diagnosed patients, early initiation of intensified antiretroviral and anti-inflammatory therapies is critical. Among viral proteins, p17 exhibits superior potential over other HIV-encoded proteins as a biomarker for early HAL risk prediction by promoting B-cell proliferation and remodeling the TME. Future directions should prioritize the establishment of p17-based dynamic risk assessment models and strategies targeting viral reservoirs to enhance early detection and optimize the management of HAL in high-risk populations.

### 10.3. EBV Viraemia

EBV has long been closely associated with HIV-PCNSL. The detection of EBV DNA in CSF demonstrates a sensitivity of 100% and a specificity of 98.5%, making it a potentially reliable diagnostic biomarker [273]. All PCNSL samples show strong positive expression of EBV-encoded small RNA (EBER1), indicating a 100% association between EBV and HIV-PCNSL [274]. Although some studies have found that EBV viremia does not serve as an independent prognostic marker and shows no significant predictive value in HIV-NHL [9,275,276,277], other research has reported that a high pretreatment plasma EBV DNA level is a poor prognostic marker in HIV-DLBCL [12,278]. Taken together, while the prognostic value of plasma EBV DNA in HIV-NHL remains controversial, its diagnostic utility in HIV-PCNSL—particularly via CSF detection—has been well established and validated.

### 10.4. KSHV Viraemia

KSHV is one of the key pathogenic factors in HIV-related malignant lymphoproliferative disorders, particularly in PEL and multicentric Castleman disease (MCD), where it has a well-established oncogenic role. PEL is almost invariably associated with KSHV infection, rendering KSHV serostatus highly specific for PEL and providing some predictive value [279,280]. Although recent advances in antiviral and immunomodulatory therapies have improved outcomes for KSHV-related MCD, patients still face a significantly elevated risk of NHL [281]. Untreated KSHV-related MCD typically follows a progressive and often fatal course, potentially evolving into PEL or KSHV-related DLBCL [282]. Such DLBCL cases may represent a clonal plasmablastic expansion driven by KSHV infection, suggesting that KSHV can mediate B-cell malignant transformation under certain pathological conditions. Moreover, in KSHV-related MCD and malignancies, KSHV frequently coexists with EBV, and the two viruses may synergistically promote tumorigenesis by modulating each other’s latent and lytic cycles, disrupting host immune responses, and altering cell fate pathways [283]. HIV infection itself contributes to this process through immunosuppression and activation of viral oncogenic mechanisms, creating a favorable microenvironment for KSHV and EBV-driven oncogenesis [283]. However, the pathogenic role of KSHV in HALs is subtype-specific. For instance, in more common HAL subtypes such as BL, DLBCL, and HL, KSHV has not been consistently detected or linked to tumor development [279,280]. Instead, these lymphomas are typically EBV-associated. Thus, while KSHV holds significant etiological and predictive relevance in PEL and certain MCD-related lymphomas, it is not suitable as a universal risk-predictive biomarker for all HAL (Table 9).

**Table 9:** Virus-related biomarkers.

Biomarker Category	Representative Biomarkers	Sample Source	Expression Trend	Clinical Relevance	References
HIV-related markers	HIV viraemia p17 *p17* *gene* *variation* Tat Nef gp120	HIV-NHL patients HIV-NHL patients/mice HIV-NHL patients HIV-NHL patients HIV-NHL patients/mice Mice	Increased Increased Increased Increased Increased Increased	Predictive/Prognostic Predictive Predictive Predictive Predictive Predictive	[131,221,223,245,246,247,248,249,250,251] [54,264,269] [270,271,272] [54,264,269] [54,264,269] [54,264,269]
Other viruses	EBV viraemia KSHV viraemia	HAL patients HIV-PEL patients	Increased Increased	Predictive/Prognostic Predictive	[9,12,275,276,277,278] [279,280,281,282,283]

Abbreviations: HAL, HIV-associated lymphoma; HIV-PEL, HIV-associated primary effusion lymphoma; Tat, transactivator of transcription; Nef, negative factor; EBV, Epstein–Barr virus; KSHV, Kaposi’s sarcoma-associated herpesvirus.

## 11. Conclusions and Perspectives

HAL remains one of the leading causes of mortality among PLWH, characterized by aggressive behavior and rapid progression. Over recent years, substantial progress has been made in the discovery of circulating biomarkers related to immune-inflammatory mediators, chemokines, soluble receptors, serum proteins, lymphocyte subsets, HIV viral parameters, and virus-encoded proteins. These biomarkers offer valuable information for early risk warning, therapeutic response monitoring, and prognostic stratification (Table 10). Dysregulated cytokine and chemokine profiles reflect immune activation and B-cell dysregulation, providing early indicators of HAL risk. Markers such as FLCs, clonal Ig DNA, β2-M, ferritin, and LDH have demonstrated potential for monitoring tumor burden and disease activity. Meanwhile, emerging biomarkers including circulating miRNAs and EVs have expanded the horizons for minimally invasive liquid biopsy applications. Lymphocyte subpopulation analysis and HIV viral load monitoring further underscore the critical role of immune function and viral persistence in HAL pathogenesis and outcomes.

Nevertheless, several challenges remain in translating these findings into clinical practice. Case number limitations, subtype heterogeneity, technical barriers, and retrospective study biases may affect biomarker validation and generalizability. Future research should prioritize large-scale, multi-center, prospective cohort studies integrating multi-omics approaches to map biomarker dynamics across HAL subtypes comprehensively. The validation of predictive models and the optimization of threshold definitions will be crucial steps toward clinical implementation. Ultimately, integrating dynamic biomarker monitoring into routine screening could enable early identification and precision intervention for high-risk PLWH. Progress in biomarker-driven strategies holds promise for transitioning HAL management toward a paradigm of dynamic surveillance and real-time intervention to significantly improve patient outcomes.

## Figures and Tables

**Table 1:** Biomarkers for cytokines

Biomarker Category	Representative Biomarkers	Sample Source	Expression Trend	Clinical Relevance	References
Cytokines	IL-1α IL-1β IL-4 IL-5 IL-6 IL-7 IL-8 IL-10 IL10 SNPs IL-11 IL-12p40 IL-13 IL-15 IL-18 IL-29 GM-CSF Neopterin TNF-α TGF-β IFN-γ BAFF/BLyS	HIV-NHL patients Mice HIV-NHL patients HIV-NHL patients HIV-NHL patients/mice HIV-NHL patients HIV-NHL patients HIV-NHL patients/mice HIV-NHL patients HIV-NHL patients Mice HIV-NHL patients/mice HIV-NHL patients HIV-NHL patients HIV-NHL patients HIV-NHL patients/mice HIV-NHL patients HIV-NHL patients/mice HIV-DLBCL patients HIV-NHL patients HIV-NHL patients	Increased Increased Increased Increased Increased Increased Increased Increased Increased Increased Increased Increased Increased Increased Increased Increased Increased Increased Increased Increased Increased	Predictive Predictive Predictive Predictive Predictive/Prognostic Prognostic Prognostic Predictive/Prognostic Predictive Predictive Predictive Predictive Prognostic Predictive Predictive Predictive Predictive Predictive Prognostic Prognostic Prognostic	[9] [54] [9] [9] [52,54,55,56,59,60,61] [62] [62] [50,52,54,55,56,59,60,61] [50,51] [7] [54] [9,54] [62] [7] [7] [9,54] [55,56] [54,55,56] [61] [62] [59,60]

Abbreviations: HIV-NHL, HIV-associated non-Hodgkin’s lymphoma; HIV-DLBC, HIV-associated Diffuse large B-cell lymphoma; IL, interleukin; SNPs, single nucleotide polymorphisms; GM-CSF, granulocyte-macrophage colony-stimulating factor; TNF-α, tumor necrosis factor alpha; TGF-β, transforming growth factor-β; IFN-γ, interferon-γ; BAFF/BlyS, B-cell-activating factor of the TNF family/B lymphocyte Stimulator.

**Table 2:** Biomarkers for chemokines

Biomarker Category	Representative Biomarkers	Sample Source	Expression Trend	Clinical Relevance	References
Chemokines	CXCL10/IP-10 CXCL11 CXCL13/BCA-1 CXCL13 SNPs CCL2 CCL8 CCL17 CCL19 MIP-1δ MIP-1β	HIV-NHL patients HIV-NHL patients HIV-NHL patients HIV-NHL patients HIV-NHL patients/mice HIV-NHL patients HIV-NHL patients HIV-NHL patients HIV-NHL patients Mice	Increased Increased Increased Increased Increased Increased Increased Increased Increased Increased	Predictive/Prognostic Predictive Predictive/Prognostic Predictive Predictive/Prognostic Predictive Predictive Predictive Predictive Predictive	[55,57,60,84] [57] [7,56,59,78,79] [56] [7,54,60] [57] [7] [57] [57] [54]

Abbreviations: HIV-NHL, HIV-associated non-Hodgkin’s lymphoma; IP-10, Interferon-inducible protein 10; BCA-1, B-cell-attracting chemokine 1; SNPs, single nucleotide polymorphisms; MIP, macrophage inflammatory protein.

**Table 3:** Biomarkers for chemokines

Biomarker Category	Representative Biomarkers	Sample Source	Expression Trend	Clinical Relevance	References
Soluble receptors	sCD23 sCD25 sCD27 sCD30 sCD163	HIV-NHL patients HIV-NHL patients HIV-NHL patients HIV-NHL patients HIV-NHL patients	Increased Increased Increased Increased Increased	Predictive Predictive/Treatment monitoring Predictive Predictive Predictive	[7,52,78,98,99,100,101] [60] [7,52,78,98,99,100] [7,52,78,98,99,100] [7,60]

Abbreviations: HIV-NHL, HIV-associated non-Hodgkin’s lymphoma; sCD, soluble cluster of differentiation.

**Table 4:** Microbial translocation biomarkers

Biomarker Category	Representative Biomarkers	Sample Source	Expression Trend	Clinical Relevance	References
Microbial translocation biomarkers	sCD14 LBP LPS FABP2 EndoCAb	HIV-NHL patients HIV-NHL patients HIV-NHL patients HIV-NHL patients HIV-NHL patients	Increased Increased Increased Increased Decreased	Predictive/Prognostic Predictive/Prognostic Predictive Predictive Predictive	[7,8,60] [7,8,60] [7,8] [7,8,60] [7,8]

Abbreviations: HIV-NHL, HIV-associated non-Hodgkin’s lymphoma; sCD, soluble cluster of differentiation; LBP, lipopolysaccharide-binding protein; LPS, lipopolysaccharides; FABP2, fatty acid-binding protein 2; EndoCAb, Endotoxin core antibody.

**Table 5:** Protein-related biomarkers.

Biomarker Category	Representative Biomarkers	Sample Source	Expression Trend	Clinical Relevance	References
Serum Protein biomarkers	κ and λ FLC Total globulin IgA IgE IgG IgM PD-1 and PD-L1 Gal-1 β2-M Ferritin LDH SAA APO C-II C4-A C1Q	HIV-NHL patients HIV-NHL patients HIV-NHL patients HIV-NHL patients HIV-NHL patients HIV-NHL patients HAL patients HIV-NHL patients HIV-NHL patients HIV-NHL patients HIV-NHL patients HIV-NHL/HL patients HIV-NHL/HL patients HIV-NHL/HL patients HIV-NHL patients	Increased Increased Unchanged Unchanged Unchanged Unchanged Increased Decreased Increased Increased Increased Increased Increased Increased Decreased	Predictive Still in dispute Unrelated Unrelated Unrelated Unrelated Predictive/Prognostic Predictive/Prognostic Predictive/Prognostic Prognostic Prognostic Predictive/Prognostic Predictive Predictive Predictive/Prognostic	[55,61,126,127,128] [130,131] [52,126,127] [52,126,127] [52,126,127] [52,126,127] [135,136] [139] [142,143] [61,150] [153,154,155,156,157,158,159,160,161,162,163,164] [167,168] [167,168] [167,168] [142]

Abbreviations: HIV-NHL, HIV-associated non-Hodgkin’s lymphoma; HAL, HIV-associated lymphoma; HIV-HL, HIV-associated Hodgkin’s lymphoma; FLC, free light chains; Ig, Immunoglobulin; Gal-1, Galectin-1; β2-M, beta 2-microglobulin; LDH, lactate dehydrogenase; SAA, serum amyloid A; APO C-II, apolipoprotein C-II; complement 4-A; C1Q, complement 1Q.

**Table 7:** Extracellular vesicle biomarkers.

Biomarker Category	Representative Biomarkers	Sample Source	Expression Trend	Clinical Relevance	References
Extracellular Vesicles	PD-L1 CD40 CD40L TNF-RII IL-6Ra	HIV-NHL patients	Increased	Predictive	[215,216]

Abbreviations: HIV-NHL, HIV-associated non-Hodgkin’s lymphoma; TNF-RII, TNF receptors; PD-L1, programmed cell death ligand 1; TNF-RII, TNF receptors-II.

**Table 8:** Lymphocyte subpopulation.

Biomarker Category	Representative Biomarkers	Sample Source	Expression Trend	Clinical Relevance	References
Lymphocyte subpopulation	CD4+T cell CD4/CD8 ratio LMR	HIV-NHL patients HIV-NHL patients HIV-DLBCL patients	Decreased Decreased Decreased	Predictive/Prognostic Predictive/Prognostic Prognostic	[131,218,219,220,221,222,223,224,225,226,227,232,233,235,236] [226,227,228,229,230,231,234,235,237] [14,240,241]

Abbreviations: HIV-NHL, HIV-associated non-Hodgkin’s lymphoma; HIV-DLBCL, HIV-associated diffuse large B-cell lymphoma; LMR, lymphocyte-to-monocyte ratio.

**Table 10:** Summary table of circulating biomarkers by clinical utility.

Clinical Utility	Type	Biomarkers	Key Features
Early Risk Prediction	Cytokines	IL-1α IL-1β IL-4 IL-5 IL-6 IL-10 IL10 SNPs IL-11 IL-12p40 IL-13 IL-18 IL-29 GM-CSF Neopterin TNF-α	Associated with immune activation and inflammation; combined monitoring of cytokine levels prior to onset of disease allows early screening of at-risk individuals.
	Chemokines	CXCL10/IP-10 CXCL11 CXCL13/BCA-1 CXCL SNPs CCL2 CCL8 CCL17 CCL19 MIP-1δ MIP-1β	Involved in B-cell migration and immunomodulation for HAL.
	Soluble receptors	sCD23 sCD27 sCD30 sCD163	Involved in the regulation of malignant B-cell pathology and associated with B-cell activation. sCD163 may also be involved in virus-driven carcinogenesis, especially in EBV-driven.
	Microbial translocation biomarkers	sCD14 LBP LPS FABP2 EndoCAb	Associated with immune activation and inflammation, also reflects the degree of pathological damage to the intestinal mucosa, the combined assay can be used as a biomarker for PLWH disease monitoring.
	Serum protein biomarkers	Κ and λ FLC PD-1 and PD-L1 Gal-1 β2-M SAA APO C-II C4-A C1Q	Responds to abnormal B-cell activation; involved in immune regulation; predicted time points may be influenced by a variety of factors; β2-M is directly associated with active tumor cell replication and increased tumor load.
	Genetic-related biomarkers	miRNA SNPs miR-17 miR-21 miR-122 miR-106a miR-106b miR-223 miR-200c-3p DNA-CNAs Clonal Ig DNA cfDNA	HIV-1 infection may exacerbate lymphoma progression and metastasis by remodeling the miRNA regulatory network and more significant correlation with risk of developing some specific lymphoma subtypes. Alterations in genes promote lymphoma development.
	Extracellular vesicles	PD-L1 CD40 CD40L TNF-RII IL-6Ra	Immunomodulatory molecules can be specifically expressed on the surface of EVs, which are involved in intercellular communication and TME regulation through ligand-receptor interactions to promote tumor progression.
	Lymphocyte subpopulation	CD4+T cell CD4/CD8 ratio	Sensitive to immune reconstitution and an important independent risk factor for predicting the development of HAL.
	Virus-related biomarkers	HIV viraemia p17 p17 gene variation Tat Nef gp120 EBV viraemia KSHV viraemia	HIV causes CD4+ T-cell depletion and chronic immune activation, indirectly increasing the risk of HAL. It can also directly increase cancer incidence through HIV-1’s own pro-cancer mechanisms. HIV proteins can drive carcinogenesis through multiple mechanisms. Synergistic carcinogenic effects of EBV and KSHV.
Prognostic Stratification	Cytokines	IL-6 IL-7 IL-8 IL-15 TGF-β IFN-γ BAFF/BLyS	Elevated levels linked to poor prognosis and disease progression; multi-cytokine models offer superior predictive performance.
	Chemokines	CXCL10/IP-10 CXCL13/BCA-1 CCL2	Elevated levels linked to poor prognosis and disease progression.
	Microbial translocation biomarkers	sCD14 LBP	Significantly negatively correlated with overall survival.
	Serum protein biomarkers	PD-1 and PD-L1 Gal-1 β2-M Ferritin LDH SAA C1Q	Indicators of poor prognosis. Gal-1 levels in serum are low, whereas in tumors Gal-1 shows relatively high expression and is associated with a better prognosis. Ferritin as an independent prognostic indicator. Elevated LDH is strongly associated with poorer PFS and OS. HAL patients with high SAA level expression also had longer survival periods. The levels of C1Q negatively correlate with HAL survival time.
	Lymphocyte subpopulation	CD4+T cell CD4/CD8 ratio LMR	Associated with shorter OS and PFS in HAL. CD4/CD8 ratio <0.5 was an independent predictor of non-AIDS-related and all-cause mortality in PLWH. Combined with IPI can further improve risk prediction and prognostic assessment accuracy.
	Virus-related biomarkers	HIV viraemia EBV viraemia	Persistent hyperviremia can increase a patient’s risk of death.
Treatment Monitoring	Cytokines	BAFF/BLyS TNF-α	Correlates with clinical response to treatment and survival.
	Chemokines	CXCL10/IP-10 CXCL13/BCA-1 CCL2 CCL17	Associated with inflammation, which partially declines or returns to normal after therapeutic intervention, with a potential role as a dynamic prognostic marker. Monitor disease progression and response to therapy.
	Soluble receptors	sCD25	Dynamic changes may reflect treatment response.
	Microbial translocation biomarkers	sCD14 LBP	Dynamic post-treatment decline may reflect reversal of partial immune activation status by treatment.
	Serum protein biomarkers	Ferritin C1Q	Ferritin levels may reflect tumor microenvironmental Status, informing treatment. C1Q decline after treatment predicts poor prognosis.
	Extracellular vesicles	PD-L1 CD40 CD40L TNF-RII	Significant reduction in plasma EVs harboring PD-L1, CD40, CD40L, or TNF-RII following treatment.
	Virus-related biomarkers	HIV viraemia	Post-treatment decline reduces risk of HAL development.

Abbreviations: IL, interleukin; SNPs, single nucleotide polymorphisms; GM-CSF, granulocyte-macrophage colony-stimulating factor; TNF-α, tumor necrosis factor alpha; IP-10, interferon-inducible protein 10; BCA-1, B-cell-attracting chemokine 1; MIP, macrophage inflammatory protein; HAL, HIV-associated lymphoma; sCD, soluble cluster of differentiation; EBV, Epstein–Barr virus; LBP, lipopolysaccharide-binding protein; LPS, lipopolysaccharides; FABP2, fatty acid-binding protein 2; EndoCAb, Endotoxin core antibody; PLWH, people living with HIV; FLC, free light chains; Ig, immunoglobulin; PD-1, Programmed cell death protein 1; PD-L1, programmed cell death ligand 1;Gal-1, galectin-1; β2-M, beta 2-microglobulin; SAA, serum amyloid A; APO C-II, apolipoprotein C-II; complement 4-A; complement 1Q; HIV, human immunodeficiency virus; miRNA, microRNA; DNA-CNAs, copy number alterations; cfDNA, circulating free DNA; TME, tumor microenvironment; TNF-RII, TNF receptors-II; Tat, transactivator of transcription; Nef, negative factor; KSHV, Kaposi’s sarcoma-associated herpesvirus; TGF-β, transforming growth factor-β; IFN-γ,interferon-γ; BAFF/BlyS, B-cell-activating factor of the TNF family/B lymphocyte Stimulator; LDH, lactate dehydrogenase; LMR, lymphocyte-to-monocyte ratio.

## Data Availability

Not applicable.
